# Application of biasing-potential replica exchange simulations for loop modeling and refinement of proteins in explicit solvent

**DOI:** 10.1186/1758-2946-3-S1-P33

**Published:** 2011-04-19

**Authors:** S Kannan, W Sippl, M Zacharias

**Affiliations:** 1 Institute of Pharmacy, Martin-Luther-Universität Halle-Wittenberg, 06120 Halle/ Saale, Germany; 2 Physics Department (T38), Technical University Munich, 85748 Garching, Germany

## 

Comparative protein modeling of a target protein based on sequence similarity to a protein with known structure is widely used to provide structural models of proteins. Frequently, the quality of the target- template sequence alignment is non-uniform along the sequence: parts can be modeled with a high confidence, whereas other parts differ strongly from the template. In principle, molecular dynamics (MD) simulations can be used to refine protein model structures and also to model loops in homology modeled protein structures, but it is limited by the currently accessible simulation time scales. In the current work we have used a recently developed biasing potential replica exchange (BP-Rex) MD [1] method to refine and to model loops in homology modeled protein structure at atomic resolution including explicit solvent. In standard Rex MD [2] simulations several replicas of a system are run in parallel at different temperatures allowing exchanges at preset time intervals. In a BP-Rex MD simulation replicas are controlled by various levels of a biasing potential to reduce the energy barriers associated with peptide backbone dihedral transitions. The method requires much fewer replicas for efficient sampling compared with standard temperature Rex MD. Starting from incorrect loop conformations this BP-Rex MD method samples the correct loop conformations as dominant conformations in all the cases. Application of BP-Rex MD to several protein loops indicates improved conformational sampling of backbone dihedral angle of loop residues compared to conventional MD simulations. BP-Rex MD refinement simulations on several test cases starting from decoy structures deviating significantly from the native structure resulted in final structures in much closer agreement with experiment compared to conventional MD simulations [3].
